# Biological effect of an alternating electric field on cell proliferation and synergistic antimitotic effect in combination with ionizing radiation

**DOI:** 10.18632/oncotarget.11407

**Published:** 2016-08-19

**Authors:** Eun Ho Kim, Ye Jin Kim, Hyo Sook Song, Youn Kyoung Jeong, Ji Young Lee, Jiwon Sung, Seung Hoon Yoo, Myonggeun Yoon

**Affiliations:** ^1^ Korea Institute of Radiological & Medical Sciences, Seoul, 01812, Korea; ^2^ Department of Bio-Convergence Engineering, Korea University, Seoul, 02841, Korea

**Keywords:** tumour-treating fields, ionizing radiation, glioblastoma multiforme, apoptosis, synergism

## Abstract

Alternating electric fields at an intermediate frequency (100~300 kHz), referred to as tumour-treating fields (TTF), are believed to interrupt the process of mitosis via apoptosis and to act as an inhibitor of cell proliferation. Although the existence of an antimitotic effect of TTF is widely known, the proposed apoptotic mechanism of TTF on cell function and the efficacy of TTF are controversial issues among medical experts. To resolve these controversial issues, a better understanding of the underlying molecular mechanisms of TTF on cell function and the differences between the effects of TTF alone and in combination with other treatment techniques is essential. Here, we report experimental evidence of TTF-induced apoptosis and the synergistic antimitotic effect of TTF in combination with ionizing radiation (IR). For these experiments, two human Glioblastoma multiforme (GBM) cells (U373 and U87) were treated either with TTF alone or with TTF followed by ionizing radiation (IR). Cell apoptosis, DNA damage, and mitotic abnormalities were quantified after the application of TTF, and their percentages were markedly increased when TTF was combined with IR. Our experimental results also suggested that TTF combined with IR synergistically suppressed both cell migration and invasion, based on the inhibition of MMP-9 and vimentin.

## INTRODUCTION

Glioblastoma multiforme (GBM) is one of the most dangerous cancers, with a poor prognosis. There is no widely recognized and effective treatment for GBM, resulting in an overall survival of 6 to 7 months under currently available optimal treatments for patients with recurrent GBM. It has been reported that a newly proposed treatment technique using alternating electric field with very low-intensity (< 2 V/cm), intermediate-frequency (100–300kHz), called tumour-treating fields (TTF), disrupts mitotic spindle formation during metaphase and can effectively inhibit the growth of a variety of human and rodent tumor cell lines [1, 2]. Recent clinical studies have shown that treating recurrent GBM patients with TTF may result in significantly longer overall survival (OS) than the standard treatment, with no unexpected adverse effects [3–7]. In contrast, the only randomized clinical trial published in the peer-reviewed literature showed that the outcome for recurrent GBM patients in the TTF group was not distinctly better than that for the conventional therapy group [8]. Recently, another randomized clinical trial in newly diagnosed glioblastoma patients has strongly suggested that the use of TTF in combination with chemotherapeutic agents improves the survival rate without a significant increase in toxicity compared with chemotherapy alone [9, 10]. Previous studies have suggested that although the clinical efficacy of TTF alone remains controversial, combination therapy with TTF and chemotherapy is more effective than chemotherapy alone for newly diagnosed GBM patients.

Although the use of TTF plus chemotherapy has been reported to improve therapeutic efficacy for GBM patients based on *in vitro* [11, 12] and clinical studies [9, 10], there have not been sufficient studies on the potential of the other treatment combinations (e.g., TTF plus ionizing radiation; TTF+IR) as an effective antitumor treatment modality. In essence, TTF is physically similar to IR in the sense that they both form regions in which an electromagnetic field occurs inside a given tissue. The difference between these two treatments is that whereas TTF acts in the near field at an intermediate frequency, IR acts in the far field region with a high frequency. In this respect, the similarities and differences between TTF and IR regarding the inhibitory effect on cell proliferation are of interest. Here, we report the underlying mechanisms of the effect of TTF with and without IR on cell function, which is necessary to increase the understanding of TTF use for better outcomes in patients.

## RESULTS AND DISCUSSIONS

### TTF-induced apoptosis

**Figure 1:**
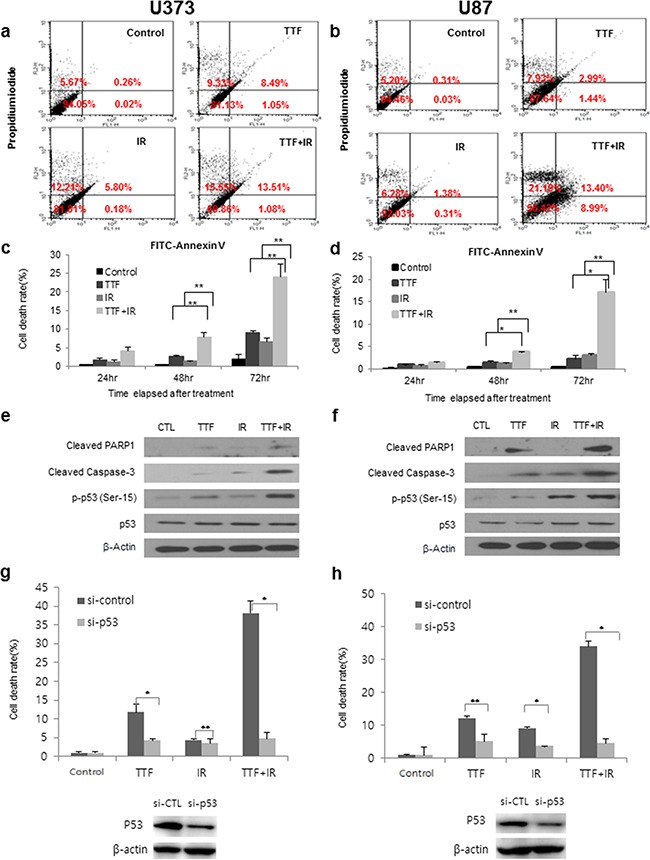

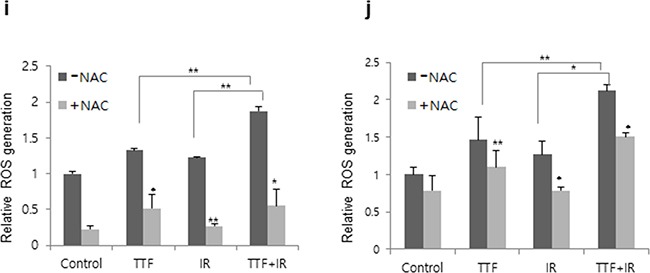
TTF induces apoptosis of GBM cells, and the effect of TTF+IR is synergistic **a, b.** Results of annexin V and PI staining after U373 and U87 cells were exposed to 72 h of TTF, 5 Gy of γ-rays or 5 Gy of γ-rays followed by 24 h of TTF, indicated as the TTF, IR and TTF+IR treatments, respectively. Percentages shown in upper left, upper right, lower left and lower right quadrants are percentages of cells showing necrosis, late apoptosis, viability, early apoptosis, respectively. **c, d.** Cell death rates measured at 24, 48 and 72 h after treatments with TTF, IR and TTF+IR. The values represent the means of three experiments ± SD; *p < 0.05, **p < 0.001. **e, f.** U373 and U87 cells were exposed to 24 h of TTF, 5 Gy of γ-rays or 5 Gy of γ-rays followed by 24 h of TTF. Immunoblotted (IB) cell lysates (30 μg) are shown with the corresponding antibodies. **g, h.** Results of annexin V and PI staining after U373 and U87 cells were transfected with siRNA (si-Ctrl, si-p53) and exposed to 24 h of TTF, 5 Gy of γ-rays or 5 Gy of γ-rays followed by 24 h of TTF, indicated as the TTF, IR and TTF+IR treatments, respectively. The values represent the means of three experiments ± SD; *p < 0.05, **p < 0.001. **i, j.** ROS rates measured at 24 h after treatments with TTF, IR and TTF+IR. The values represent the means of three experiments ± SD; *p < 0.05, **p < 0.001.

To clarify the induction of apoptosis, we assessed early apoptosis by using Annexin V-FITC/PI flow cytometry. Figure 1a-1b show the results of Annexin V-FITC/PI flow cytometry for the control, TTF-treated cells, IR-treated cells and TTF+IR-treated cells in two GBM cell lines. As seen in Figure 1a-1b, TTF significantly increased the percentage of early apoptotic cells in both glioblastoma cell lines, which is generally observed in IR-treated cell lines [1]. For quantitative analysis of the synergistic effect of TTF+IR on cell function depending on time of cell harvesting, cell death rates were measured at 24, 48 and 72 h after all of the treatments were complete. The combination of Annexin V-FITC and propidium iodide means the distinction between early apoptotic cells (Annexin V-FITC positive), late apoptotic and/or necrotic cells (Annexin V-FITC and propidium iodide positive), and viable cells (unstained). The percentage of cell death in U373 cells (U87) at 72 h after TTF+IR treatment was 23.9 (17.1) %, which was higher than the sum of the percentages of cell death resulting from either TTF or IR alone measured at 72 h after each treatment, which was 9.10 (2.09) % or 6.54 (2.98) % (Figure 1c-1d). Here, the cell death rate was defined as a ratio of apoptotic and/or necrotic cells to total cells counted. The results also showed that the cell death rates were increased as the time elapsed after TTF application. This residual effect was reported previously when TTF + chemotherapeutic treatments were applied to human breast carcinoma and human glioma [12]. Although the values were different, the results were similar when cell death rates were measured at 24 and 48 h after the treatments. These experimental results regarding the effects of TTF, IR and TTF+IR on GBM cells suggest that TTF induces apoptosis of GBM cells and that the effect of TTF+IR is synergistic.

We also examined whether TTF alone enhanced the cytotoxicity resulting from the additional activation of caspase, which is a unique characteristic of apoptotic cell death. In addition, caspase-3 activation leads to cleavage of PARP-1, which can be detected in western blots by the appearance of a distinct band at 89 kDa, indicating that cleaved PARP-1 is present [13]. As seen in Figure 1e-1f, enhanced PARP and caspase-3 cleavage was observed after treatment with TTF. Examination of the level of phosphorylated p53, which was performed to analyse the effect of p53 on TTF-induced apoptosis, showed that the p53 tumour suppressor acted to integrate multiple stress signals into a series of diverse antiproliferative responses. Because one of the most important functions of p53 is its ability to activate apoptosis, disruption of this process can promote tumour progression and chemoresistance. Therefore, we further analysed the dependent signalling effect of p53 on TTF-induced apoptosis. Phosphorylation of p53 plays important roles in regulating the biological activities of p53[14], and the phosphorylation of p53 at Ser-15 and Ser-20 is involved in activating p53. We found that enhanced levels of p-p53 (Ser-15) were again detected after TTF treatment (Figure 1e-1f), indicating that TTF-induced apoptosis involves the p53-dependent pathway. In addition to the cellular effects of TTF alone, we examined the combinatorial effect of TTF+IR on cell function. The analysis showed that PARP and caspase-3 cleavage was synergistically enhanced by treatment with TTF+IR compared with treatment with TTF or IR alone (Figure 1e-1f). To further investigate the p53 dependency effect of apoptosis with TTF in combination with IR, the level of phosphorylated p53 was also examined, and the results showed that, similarly to the results of the PARP and caspase-3 cleavage experiment, synergistic effects on the levels of p-p53 (Ser-15) occurred after TTF+IR treatment (Figure 1e-1f). To confirm the relationship between p53 and cytotoxicity after the treatment of TTF+IR on GBM cells, we tested the cell death on the p53 knockdown cells. Cells were transfected with p53 siRNA for 24 h and treated with TTF, IR and TTF+IR for another 48 h. As shown in Figure 1g-1h, compared to control group, the cell viability of p53 knockdown groups was decreased after each treatment. These results suggest that TTF+IR induced apoptosis may act in a p53-dependent manner. In conclusion, apoptosis induced by TTF were through subsequent activation of caspase pathway in a p53-dependent manner. To investigate the relationship between production of reactive oxygen species (ROS) and enhancement of radiation-induced apoptosis by TTF, we examined the effects of TTF on ROS production in GBM cells. ROS production was induced more by the combination of TTF and radiation than by TTF or radiation alone. These data were further confirmed by incubating the cells with NAC, which is a scavenger of ROS and treatment with NAC almost completely decreased the release of ROS in GBM cells (Figure 1i-1j).

### The effect of TTF on cell function: TTF vs. IR vs. TTF+IR

**Figure 2:**
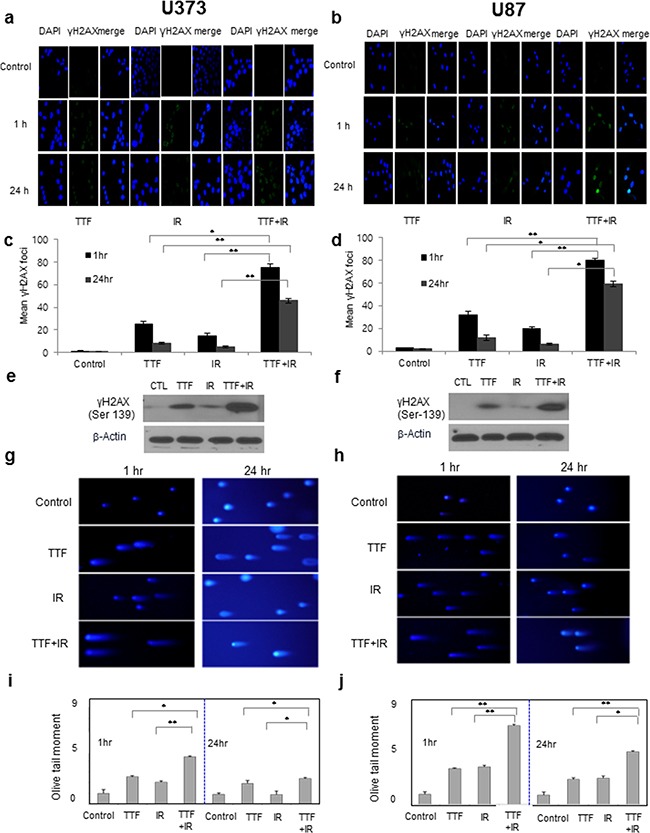
The effect of TTF on cell function is similar to that of IR, and the effect of TTF+IR is synergistic Cells were exposed to 24 h of TTF, 5 Gy of γ-rays or 5 Gy of γ-rays followed by 24 h of TTF, indicated as the TTF, IR and TTF+IR treatments, respectively. **a, b.** Immunocytochemistry staining for H2AX phosphorylation (Ser 139, green) in U373 and U87 cells, measured at 1 h and 24 h after treatment with TTF, IR or TTF+IR. **c, d.** The mean and standard error of the calculated γ-H2AX foci from 3 experiments measured at 1 h and 24 h after treatment with TTF, IR and TTF+IR. The values represent the means of three experiments ± SD; *p < 0.05, **p < 0.001. **e, f.** Cell lysates immunoblotted with the indicated antibodies after TTF, measured at 24 h after treatment with TTF, IR or TTF+IR. **g, h.** Neutral comet assay in the U373 and U87 cell lines, measured at 1 h and 24 h after treatment with TTF, IR or TTF+IR. **i, j.** Quantitative analysis of tail movements, measured at 1 h and 24 h after treatment with TTF, IR or TTF+IR. The values represent the means of three experiments ± SD; *p < 0.05, **p < 0.001.

IR is known to result in DNA damage, including double-strand breaks (DSBs) in primary lethal lesions [15], which in turn initiate a variety of signalling events in cancer cells. DSBs lead to the phosphorylation of H2AX, which has been used as a marker of the cellular response to radiation-induced DNA damage [16]. To investigate the similarities and differences between TTF and IR regarding the effects against DNA damage, we examined the TTF-induced DNA damage response and repair processing based on the expression of the DSB marker protein phosphorylated H2AX by using immunofluorescence and western blot assays. Similarly to the results of IR treatment, both glioblastoma cell lines treated with TTF alone exhibited damaged DNA foci (i.e., DNA fragmentation), and the number of DNA foci were enhanced by the TTF+IR treatment (Figure 2a-2b). Interestingly, whereas damaged DNA had disappeared by 24 h after IR exposure without TTF, prolonged expression of γ-H2AX was observed 24 h after IR radiation exposure in the presence of TTF. Quantitative analysis of the synergistic effect of TTF+IR on the expression of γ-H2AX was performed based on the mean percentage of γ-H2AX foci in the two GBM cell lines. The mean percentages of γ-H2AX foci in the control, TTF alone, IR alone and TTF+IR treatment groups were 1(1)%, 25 (8)%, 15 (5)%, and 75 (46)% in U373 cells and 3 (2.1)% 32 (12)%, 20 (6)%, and 80 (59)% in U87 cells, respectively, when measured 1 h (24 h) after each treatment. These data again suggested that TTF+IR had a synergistic effect on DNA damage in GBM cells (Figure 2c-2d).

Western blot analysis also showed that TTF combined with IR markedly enhanced DNA damage compared with either TTF or IR alone through prolonged increased levels of γ-H2AX (Figure 2e-2f). To determine whether TTF treatment is another primary reason for high levels of DNA damage, GBM cells were exposed to IR with and without TTF, and DNA damage was measured by using the neutral comet assay. As shown in Figure 2g-2h, DNA damage was observed in both GBM cell lines, as indicated by long “comet tails”, when TTF alone was applied. The quantitative analysis showed that the olive tail movements measured in the control, TTF alone, IR alone and TTF+IR groups at 1 h (24 h) after each treatment were 1.00 (0.91)/1.00 (0.91), 2.56 (1.94)/3.48 (2.43), 2.05 (0.87)/3.65 (2.54) and 4.42 (2.38)/7.68 (5.13) in U373/U87 cells, respectively (Figure 2i-2j). The results of the neutral comet assay demonstrated that TTF increased radiation-induced DSBs, suggesting that TTF inhibits the repair of radiation-induced DSBs and that the effect of TTF+IR treatment on DNA damage is synergistic.

### Synergistic effect of TTF plus radiation for the induction of multinucleation and mitotic abnormalities in glioblastoma cells

**Figure 3:**
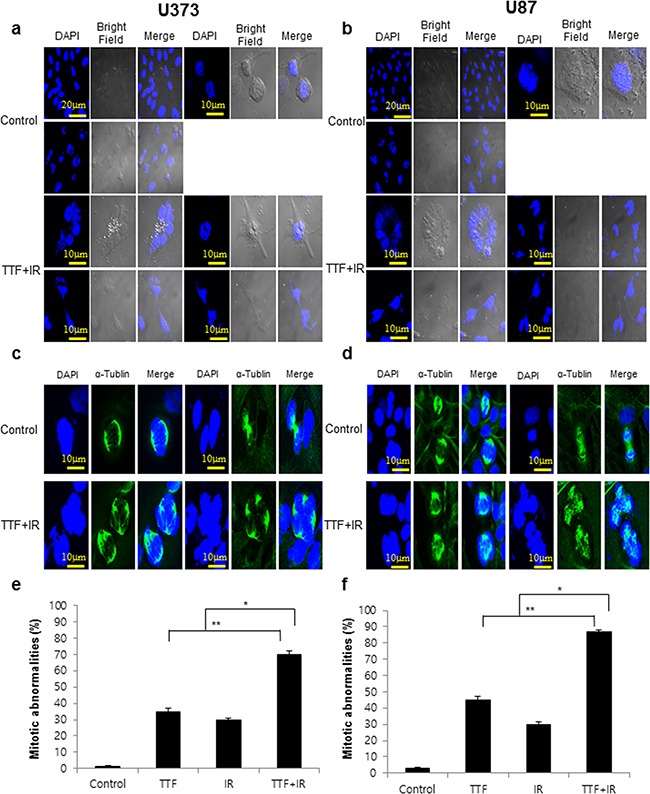
TTF+IR triggers multinucleation and mitotic abnormalities in glioblastoma cells Cells were exposed to 24 h of TTF, 5 Gy of γ-rays or 5 Gy of γ-rays followed by 24 h of TTF, indicated as the TTF, IR and TTF+IR treatments, respectively. **a, b.** Glioblastoma cells treated with TTF+IR. **c, d.** Immunofluorescence microscopy image of cells stained for α-tubulin (green) and DAPI. **e, f.** The histograms summarize the results of three independent experiments (with at least 100 cells counted in each experiment in each column). The values represent the means of three experiments ± SD; *p < 0.05, **p < 0.001. Cells were scored for the presence (abnormal) or absence (normal) of chromosome alignment and segregation defects. The image was acquired 24 h after the treatment was complete.

During mitosis, proliferating cells undergo several structural and molecular changes characterized by chromatin condensation, spindle formation, nuclear envelope fragmentation and cytoskeleton reorganization. Chromosome segregation is carried out by the complex machinery of the mitotic spindle, which is composed of a bipolar array of microtubules. The rate of microtubule disassembly from a state of polymerization to depolymerization is referred to as the catastrophe rate. Mitotic catastrophe (MC) has long been considered as a mode of cell death that results from premature or inappropriate entry of cells into mitosis and can be caused by chemical or IR-induced physical stresses. Similarly to IR treatments, which cause unbalanced MC and induce mitotic abnormalities, TTF treatment has also been reported to result in aberrant mitotic spindle formations [1, 2]. The effect of TTF+IR appeared to cause an increased proportion of cells with a multi-nucleation phenotype, which may be due to the results of dis-regulated cytokinesis (Figure 3a-3b). In the presence of TTF+IR, GBM cells assembled both mono- and multipolar spindle structures (Figure 3c-3d) that did not support proper cell division. TTF+IR treatment significantly increased the fraction of cells undergoing mitotic catastrophe, as indicated by the presence of abnormal multi-lobulated nuclei, suggesting a synergistic effect of TTF+IR treatment on cell death and the DNA damage response (Figure 3e-3f).

### The effect of TTF on the cell cycle and the invasion or migration of GBM cells: TTF vs. IR vs. TTF+IR

**Figure 4:**
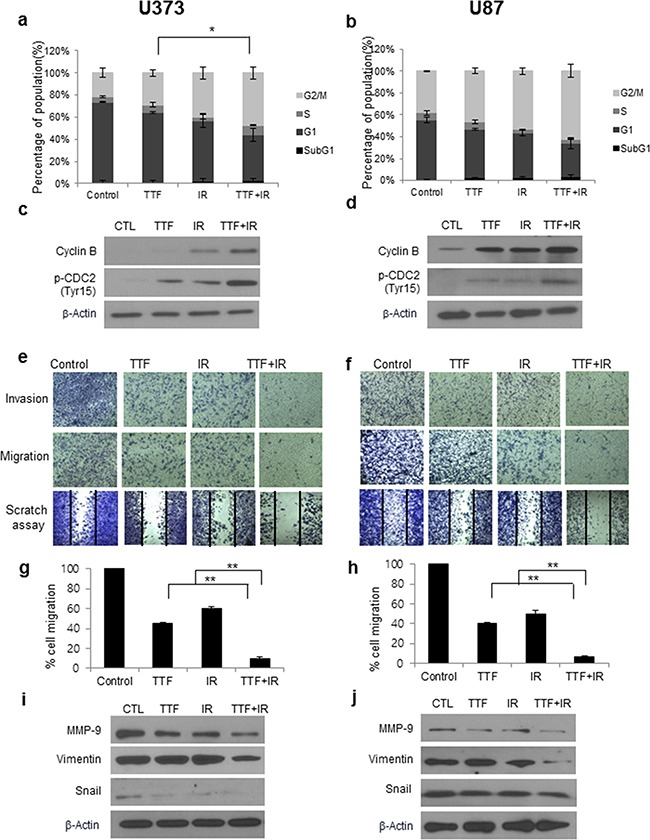
The effect of treatment with TTF and IR on the cell cycle and the invasion or migration of GBM cells Cells were exposed to 24 h of TTF, 5 Gy of γ-rays or 5 Gy of γ-rays followed by 24 h of TTF, indicated as the TTF, IR and TTF+IR treatments, respectively. **a, b.** The cell cycle distribution in U373 and U87 cells at 24 h after treatment with TTF, IR or TTF+IR. The values represent the means of three experiments ± SD; *p < 0.05, **p < 0.001. **c, d.** Cell lysates of TTF immunoblotted with the indicated antibodies, measured 24 h after treatment with TTF, IR or TTF+IR. **e, f.** Tumour cell invasion and migration were measured in a transwell assay. The numbers of tumour cells that invaded through the Matrigel (upper) and gelatine (middle) were counted in 5 high-power fields. GBM cells were treated with TTF with and without IR, and after incubation for 24 h, the cells were scraped with yellow tips to perform scratch assays (lower). **g, h.** The number of cells that migrated across the wound is shown. Each assay was photographed, based on which the distances between the migrating cell edges were quantified, and the percentage of cell migration was calculated. The values represent the means of three experiments ± SD; *p < 0.05, **p < 0.001. **i, j.** Immunoblotted (IB) cell lysates (30 μg) are shown with the corresponding antibodies.

Figure 4a-4b shows cell cycle profiles resulting from treatment with TTF alone, IR alone or TTF+IR. The results indicate that TTF+IR caused substantial accumulation of cells in subG1 phase, indicating an induction of cell cycle arrest at G2/M phase in both GBM cell lines. Quantitative analysis showed that the increase in the population due to TTF+IR treatment was larger than the sum of the increase in the population resulting from TTF alone and IR alone. We also examined the expression of a cell cycle regulator following combined treatment with TTF and radiation. Western blotting showed that radiation and TTF alone led to significant accumulation of cyclin B and p-CDC2, a key cell cycle regulator involved in the G2/M transition (Figure 4c-4d). Combined TTF and radiation treatment increased the radiation-induced accumulation of cyclin B and p-CDC2. It has been reported that TTF treatment of GBM cells increases G2/M arrest, causing an increased cell population in sub-G1 phase [17], and radiosensitive cell lines undergo a longer delay in G2/M phase compared with IR–resistant cell lines [18]. Therefore, one possible mechanism for the synergistic effect of TTF+IR treatment may be related to a correlation between radiosensitivity and the G2/M phase delay. Thus, the increase in G2/M arrest due to TTF synergistically results in PARP and caspase-3 activation, ultimately increasing cellular apoptosis.

We next evaluated the effects of TTF on the invasive and migratory capacities of GBM cells by using a scratch assay and a transwell chamber migration assay. The migration of cancer cells is an important event in the metastatic cascade of cancers, because cancer cell migration and invasion into adjacent tissues and intravasation into blood/lymphatic vessels should be minimized to prevent the metastasis of common human cancers. The experimental results showed that TTF alone inhibited cell migration, and the inhibitory effect was significantly increased by combinatorial treatment with TTF+IR (Figure 4e-4f). The transwell invasion assay indicated that IR combined with TTF was highly effective in inhibiting tumour cell invasion in both cell lines; the percentages of cell migration recorded in the U373 (U87) cell line were 45 (40)%, 60 (52)%, 10 (7)% for treatment with TTF alone, IR alone and TTF+IR, respectively, suggesting that the effect of TTF+IR is synergistic (Figure 4g-4h).

To determine the proteins involved in the inhibition of invasion induced by TTF and IR, we evaluated the expression of proteins involved in epithelial–mesenchymal transition (EMT) via western blot analysis. Tumour metastasis is a multistep process through which tumour cells disseminate from their primary site and form secondary tumours at a distant site. Metastasis occurs through multiple steps, including local invasion, intravasation, transport, extravasation, and colonization. The EMT developmental program has been shown to play a critical role in promoting metastasis in carcinoma. Vimentin is widely used as a marker of EMT that occurs during metastasis. Matrix metalloproteinase-9 (MMP-9) expression is also known to enhance the invasion and metastasis of tumour cells. The potential functions of MMP-9 include inhibition of cancer progression, activation of angiogenesis, and recruitment of macrophages or other bone marrow–derived myeloid cells to the pre-existing metastatic niche [19] and is useful for preventing metastasis in cancer patients [20]. As shown in Figure 4i-4j, TTF+IR inhibited the expression of proteins involved in this process, such as vimentin, and suppressed the expression of MMP-9, which would lead to invasion and decreased the level of EMT transcription factor snail. The results showed that the effects were significantly enhanced by the application of TTF+IR. Additionally, our experimental results showed that TTF itself significantly suppressed the invasive and migratory capacities of GBM cells, and its effect was synergistically increased by combinatorial treatment with TTF+IR.

**Figure 5:**
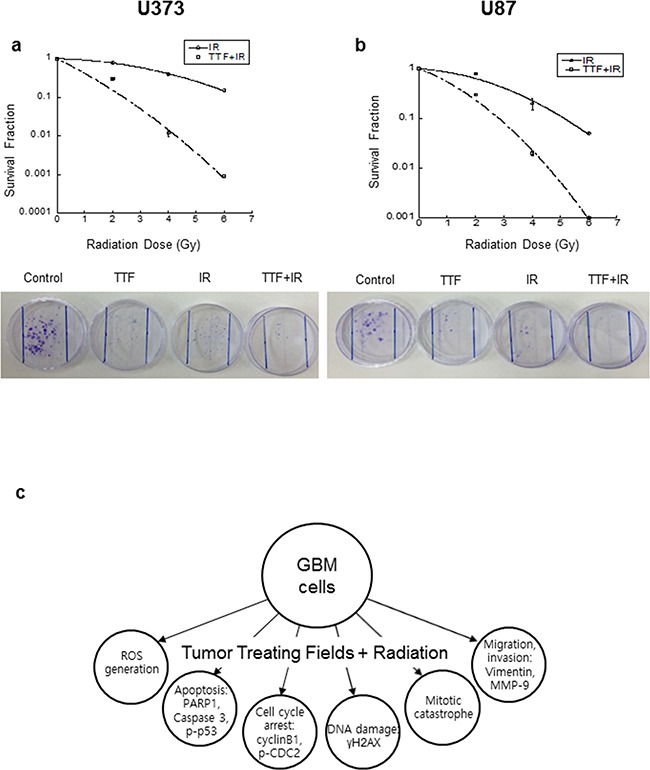
**a, b.** Cell survival curves for the U373 and U87 cell lines, with and without 48 h of TTF pretreatment, followed by various doses of γ-rays, as measured in a colony forming assay. The values represent the means of three experiments ± SD. **c.** The proposed mechanisms for TTF as a radiosensitiser of glioblastoma cell.

Currently, radiation therapy is considered only as an adjuvant therapy for patients with newly diagnosed GBM and should be carried out as a standard of care f1ollowing surgery [21, 22]. GBM tumours contain zones of tissue exhibiting hypoxia, making them a highly radio-resistant type of tumour. Although various approaches have been pursued to improve radio-sensitivity, there has been only limited success to date, which may be one of the reasons that radiotherapy alone is not the standard of care for patients with newly diagnosed GBM. To evaluate whether TTF+IR overcomes the limitations of IR alone for patients with newly diagnosed GBM, we carried out a colony forming assay by increasing the radiation dose from 2 Gy to 6 Gy after 24 h of TTF exposure. The results of the colony forming assays in the γ-irradiated GBM cells with and without TTF pretreatment, presented in the form of survival curves, are shown in Figure 5a-5b. The interpolated values from the fitted curves revealed a significant decrease in the survival fraction induced by IR combined with TTF pretreatment in comparison with IR alone. Parameters from linear quadratic fitting of survival curves, the dose required to reduce survival to 10%, and % of cells in apoptosis, are listed in Table 1, Table 2, Table 3, and Table 4. This result suggests that radiotherapy with TTF pretreatment may be considered as a strong candidate for significantly improving clinical outcomes. The proposed mechanisms for TTF as a radiosensitiser of glioblastoma cell were shown in Figure 5c. Because the radiosensitizing effects of TTF are intended to enhance tumour cell death, while having a much less of an effect on normal tissues, experiments with i*n vivo* mouse models and normal tissue should also be conducted in future studies to minimize the possible complications in clinical applications.

**Table 1:** Linear quadratic fitting parameters α and β for survival curves in cells irradiated with or without TTF

Cell type	Treatment	α (Gy^−1^)	β (Gy^−1^)
	γ-ray	0.031 ± 0.571	0.048 ± 0.108
U373	γ-ray+TTF	0.806 ± 0.655	0.063 ± 0.127
U87	γ-ray	0.088 ± 0.599	0.070 ± 0.115
	γ-ray+TTF	0.507 ± 0.601	0.109 ± 0.116

**Table 2:** Radiation dose (Gy) required with and without TTF to kill 90% of cells. values were obtained from Figure 5a-5b

Cell type	Radiation	TTF	TTF
U373	γ-ray	6.61	2.40
U87	γ-ray	5.14	2.33

**Table 3:** Radiosensitivity enhancement factor (REF) and dose reduction. REF is the ratio of the dose required with or without TTF to kill 90% of cells

Cell type	Radiation	REF	Dose reduction (%)
U373	γ-ray	2.75	64
U87	γ-ray	2.21	55

**Table 4:** Evaluation of the radiosensitization effect of TTF according to valeriote and carpentier with formula

Cell type	Radiation	SF_TTF+IR_	SF_TTF_XSF_IR_	Radiosensitization effect
U373	2Gy	0.460	0.476	Synergism
	4Gy	0.097	0.117	Synergism
	6Gy	0.010	0.047	Synergism
U87	2Gy	0.310	0.560	Synergism
	4Gy	0.021	0.140	Synergism
	6Gy	0.001	0.035	Synergism

## CONCLUSIONS

In conclusion, our investigation obtained clear evidence that TTF induces apoptosis through increased ROS and that the synergistic antimitotic effects of TTF+IR treatment increase mitotic catastrophe and tumour cell invasiveness in GBM cells via inhibition of cell survival, cell cycle regulation, and DNA repair activity. We believe that these results provide biological evidence of the molecular basis of TTF combined with IR for use in the treatment of GBM, and this work further provides a preclinical basis for the application of TTF in combination with radiotherapy in the clinic.

## MATERIALS AND METHODS

### Experimental setup for electric field and radiation

Very low-intensity (< 2 V/cm), intermediate-frequency (100–300kHz), alternating electric fields induced by insulated electrodes was reported to inhibit the growth of various tumour cells and named tumour-treating fields (TTF) [1].In this experiment, TTF was generated with a pair of insulated wires connected to a function generator and a high-voltage amplifier that generated sine-wave signals ranging from 0 to 800 V. The applied electric field intensity and frequency were 0.9 V/cm and 150 kHz, respectively. The reason why we chose 0.9V/cm as a field intensity was it is very close to the field intensity which is currently used in clinic. For irradiation, cells were plated in 60 mm dishes and incubated at 37°C under humidified conditions and 5% CO_2_ to 70–80% confluence. The cells were then irradiated with a ^137^Cs γ-ray source (Atomic Energy of Canada, Ltd., Ontario, Canada) at a dose rate of 3.81 Gy/min. We used the MTT assay to determine the optimal TTF conditions, and the experimental conditions of 0.9 V/cm and 150 kHz resulted in an inhibitory concentration of ~30% after 24 hours (h) of exposure. In our experiment involving combination treatment with TTF+IR, TTF was applied first, and IR was applied immediately after the TTF treatment was complete.

### Antibodies and chemicals

Anti-b–actin, anti-p53, anti-cyclin B, anti-vimentin and anti-Snail were purchased from Santa Cruz Biotechnology (Dallas, Texas USA). Anti-cleaved poly(ADP-ribose) polymerase-1 (PARP1), anti-phospho p53, anti-cleaved caspase 3, anti-phospho CDC2 and anti-MMP9 antibodies were obtained from Cell Signaling Technology (Danvers, MA, USA), and anti-phosphorylated H2AX (γH2AX) was obtained from Millipore (Billerica, MA, USA).

### Cell culture

Human glioblastoma U87 cell and Human glioblastoma U373 cell were obtained from the Korean Cell Line Bank (Seoul, South Korea). U87 cells were grown in MEM supplemented with 10% FBS, glutamine, HEPES, and antibiotics at 37°C in a humidified incubator under 5% CO_2_. U373 cells were grown in RPMI 1640 medium supplemented with 10% festal bovine serum (FBS), glutamine, HEPES, and antibiotics at 37°C in a humidified incubator under 5% CO_2_.

### Colony-forming assay

Cells were treated with TTF 24 h before radiation exposure and then incubated. After 14–20 days, colonies were stained with 0.4% crystal violet (Sigma, St. Louis, MO, USA). The plating efficiency (PE) represents the percentage of seeded cells that grew into colonies under the specific culture conditions of a given cell line. The survival fraction, expressed as a function of irradiation, was calculated as follows: survival fraction = colonies counted/(cells seeded × PE/100). The radiosensitization effect of TTF is evaluated according to Valeriote and Carpentier with formula.

Synergism: SF_IR+TTF_ <SF_IR_X SF_TTF_

Additivity: SF_IR+TTF_=SF_IR_X SF_TTF_

Where SF = survival fraction.

### Flow cytometry

Cells were cultured, harvested at the indicated times, and stained with propidium iodide (1 μg/mL, Sigma) according to the manufacturer's protocol, then analysed using a FACScan flow cytometer (Becton Dickinson, Franklin Lakes, NJ, USA). A minimum of 10,000 cells was counted for each sample, and data analysis was performed with CellQuest software (BD Biosciences).

### Detection of apoptotic cells through annexin V staining

After TTF exposure for 24 h, the cells were treated with IR and then incubated for a further 24 h, 48 h and 72 hr. The cells were subsequently washed with ice-cold phosphate buffered saline (PBS), trypsinized, and re-suspended in 1× binding buffer (10 mm HEPES/NaOH [pH 7.4], 140 mm NaCl, and 2.5=mm CaCl_2_) at 1 × 10^6^ cells/mL. Aliquots (100 μL) of the cell solution were mixed with 5 μL of annexin V-FITC (PharMingen) and 10 μL of a propidium iodide stock solution (50 μg/mL in PBS) via gentle vortexing, followed by 15 minutes of incubation at room temperature in the dark. Buffer (400 μL, 1×) was added to each sample, which was then analysed on a FACScan flow cytometer (Becton Dickinson, Franklin Lakes, NJ, USA). A minimum of 10,000 cells was counted for each sample, and data analysis was performed using CellQuest software (BD Biosciences).

### Intracellular reactive oxygen species detection

ROS in monocytes were monitored using the fluorescent ROS indicator, C2′,7′-dichlorodihydrofluorescein diacetate (H_2_DCFDA 5 μM; Molecular Probes). Cell-associated fluorescence was detected by FACS using a FACSort™ flow cytometer with CellQuest™ software (BD Biosciences).

### Immunocytochemistry

Immunocytochemistry was performed to determine the nuclear distribution of γ-H2AX in individual cells. Cells were grown on chambered slides 1 day prior to TTF treatment. After TTF exposure for 24 h, IR was administered to the cells, and the cells were treated for various lengths of time. All treatments were performed while the cells remained attached to the slides, followed by fixation with 4% paraformaldehyde and permeabilization with 0.2% Triton X-100 in PBS. Detection was performed after blocking the slides in 10% FBS/1% bovine serum albumin for 1 hour with a 1:1,000 dilution of a FITC-labelled mouse monoclonal antibody against γ-H2AX (Millipore, Billerica, MA, USA).

### Neutral comet assay

To detect double-strand breaks (DSBs), a neutral comet (single-cell gel electrophoresis) assay was performed according to the manufacturer's instructions (Trevigen, Gaithersburg, MD). Cells were plated in 100 mm tissue culture dishes at 1 × 10^6^ cells/dish and incubated overnight. After TTF exposure for 24 h, the cells were irradiated and incubated for 1 or 24 h. The cells were then immediately lysed at 4°C for 1 h in lysis buffer (2.5 M NaCl, 100 mM ethylenediamine tetraacetic acid, 10 mM Tris-HCl, 1% N-lauroylsarcosine, 1% Triton X-100, 10% DMSO, pH 10.0) and subjected to neutral electrophoresis buffer at 4°C. To detect DNA, the slides were stained with ethidium bromide and examined for fluorescence emission using a 515–560 nm excitation filter and a 590 nm barrier filter. DNA damage was quantified through computer-assisted image analysis (Komet analysis software, ver. 3.1; Kinetic Imaging, Liverpool, UK) to integrate fluorescence intensity.

### Western blotting

After TTF for 24 h, the glioblastoma cells were treated with IR and then incubated for 1 h or 24 h. The cells were subsequently lysed with RIPA buffer, and proteins were separated via sodium-polyacrylamide gel electrophoresis and transferred to nitrocellulose membranes. The membranes were blocked with 1% (v/v) non-fat dried milk in Tris-buffered saline with 0.05% Tween 20 and incubated with the required antibodies. Primary antibodies were used at a 1:1,000 dilution (5% bovine serum albumin) and secondary antibodies at a 1:5,000 dilution (5% skim milk). Immunoreactive protein bands were visualized via enhanced chemiluminescence (Amersham Biosciences) and scanned.

### Wound healing (scratch) assay

The cell motility assay was conducted in 6-well plates (Corning). A fine scratch in the form of a groove was made using a sterile pipette tip in a layer of cells at a confluency of approximately 90%. The cells were then treated with either TTF or radiation, or with a combination of both. Cell migration was monitored using an Eclipse Ti microscope with a DS-Fi1 camera (Nikon, Tokyo, Japan). The cells were counted using ImageJ (US National Institutes of Health).

### Invasion assay

The invasive ability was measured *in vitro* using transwell chambers, according to the manufacturer's protocol. Briefly, cells were seeded onto the membrane of the upper chamber of the transwell at a concentration of 4 × 10^5^ cells/mL in 150 μL of medium and were left untreated or were treated with TTF, radiation, or a combination of both for 24 hours. The medium in the upper chamber was serum-free, while the medium in the lower chamber contained 10% FBS as a source of chemo-attractants. Cells that passed through the Matrigel-coated membrane were stained with the Cell Stain Solution containing crystal violet supplied in the transwell invasion assay (Chemicon, Millipore, GA) and were photographed after 24 hours of incubation.

### Statistical analysis

Statistical significance was determined by Student's t-test. Differences were considered significant if the p value was less than 0.05 or 0.001.
